# N-acetylcysteine protects septic acute kidney injury by inhibiting SIRT3-mediated mitochondrial dysfunction and apoptosis

**DOI:** 10.22038/IJBMS.2024.72882.15853

**Published:** 2024

**Authors:** Heng Fan, Jian-wei Le, Min Sun, Jian-hua Zhu

**Affiliations:** 1 Department of Intensive Care Unit, The First Affiliated Hospital of Ningbo University, Ningbo, Zhejiang Province, P.R China

**Keywords:** Acute kidney injury, Apoptosis, Mitochondrial dysfunction, N-acetylcysteine, Sepsis, SIRT3

## Abstract

**Objective(s):**

To investigate the protective effect of N-acetylcysteine (NAC) on septic acute kidney injury (SAKI) via regulating Sirtuin3 (SIRT3)-mediated mitochondrial dysfunction and apoptosis.

**Materials and Methods:**

By constructing SIRT3 knockout mice and culturing kidney tubular epithelial cells (KTECs), we assessed the changes of renal function and detected the protein expression of adenine nucleotide translocator (ANT), cyclophilin (CypD) and voltage-dependent anion channel (VDAC) using western-blotting, and simultaneously detected toll-like receptor 4 (TLR4), inhibitor of kappa B kinase (IKKβ), inhibitor of Kappa Bα (IκBα), and p65 protein expression. We observed mitochondrial damage of KTECs using a transmission electron microscope and assessed apoptosis by TdT-mediated dUTP Nick-End Labeling and flow cytometry.

**Results:**

SIRT3 deficiency led to the deterioration of renal function, and caused a significant increase in inducible nitric oxide synthase production, a decrease in mitochondrial volume, up-regulation of TLR4, IκBα, IKKβ, and p65 proteins, and up-regulation of ANT, CypD and VDAC proteins. However, NAC significantly improved renal function and down-regulated the expression of TLR4, IκBα, IKKβ, and p65 proteins. Furthermore, SIRT3 deficiency led to a significant increase in KTEC apoptosis, while NAC up-regulated the expression of SIRT3 and inhibited apoptosis.

**Conclusion:**

NAC has a significant protective effect on SAKI by inhibiting SIRT3-mediated mitochondrial dysfunction and apoptosis of KTECs.

## Introduction

Acute kidney injury (AKI) is an acute disease with a sudden decline in renal function, which is commonly caused by ischemia-reperfusion injury, nephrotoxicity, and severe infection (sepsis) (1). According to statistics, AKI incidence in ordinary hospitalized patients is about 7%, while the incidence in the intensive care unit (ICU) is 25%, and the resulting mortality rate is 50% to 80% (2). AKI involves changes in the morphology and function of kidney tubular epithelial cells (KTECs), which trigger lymphocytes, macrophages, natural killer cells, and neutrophils to infiltrate the kidney, and cause KTECs to release inflammatory factors (3). When the inflammation is further aggravated, related immune factors are activated, producing inflammasomes, triggering the release of cytokines, and inducing cell necrosis or pyrolysis (4).

The characteristic of AKI is to damage KTECs rich in mitochondria, so the morphological and functional changes of mitochondria are important signs of AKI *(*5). Increased reactive oxygen species (ROS) is a reminder of decreased anti-oxidant defense capacity and also an important sign of mitochondrial damage (6). Sirtuin3 (SIRT3), as an important deacetylase in cells, is the main regulator of mitochondrial function (7). SIRT3 often exists in the form of an inactive protein and is activated in mitochondria. Increased expression of SIRT3 significantly reduces intracellular ROS and improves mitochondrial function (8). It can be seen that SIRT3 is an important regulatory factor for the repair of KTECs in the AKI process.

The pathophysiological mechanism of AKI is complex, and severe infection is its common cause. The pathogenesis of septic acute kidney injury (SAKI) usually involves inflammation, oxidative stress, and mitochondrial dysfunction in KTECs (9). In the present study, we used SIRT3 knockout (KO) mice models and KTECs to investigate SIRT3 on SAKI-related mitochondrial damage and to clarify the effect of SIRT3 on toll-like receptor 4 (TLR4)-nuclear factor-kappa B (NF-κB) signaling pathway and explore the potential therapeutic effects of N-acetylcysteine (NAC) on SAKI.

## Materials and Methods

***Animals and grouping***

C57BL/6 mice (Suzhou, Soochow University, China), male, 10–12 weeks old, 25–30g weight, were raised in Ningbo University. Wild-type (WT) and 129sv-SIRT3 KO litters (Jackson Laboratory, Shanghai, China) were used in the experiment. We randomly divided the C57BL/6 mice into the wild-type (WT)+sham, WT+cecal ligation and perforation (CLP), WT+CLP+NAC, KO+sham, KO+CLP, and KO+CLP+NAC group. We used the CLP method to construct a sepsis model (10). The specific steps: anesthetized the mice with isoflurane, made a midline incision (2 cm) in the midline of the abdomen, ligated suture at the colon and cecum junction with 5-0 silk, punctured the cecum thrice with a needle, and sutured the incision with 5-0 fine thread. The sham group was performed with the same methods as CLP but without CLP. For the NAC groups, the mice were pretreated with anti-oxidant NAC (200 mg/kg) intraperitoneally for 3 days, and 12 hr after the last pretreatment received CLP (4). Twenty four hours after the CLP, we collected blood, centrifuged it at 3500×g for 12–15 min, and stored it in a -80 °C refrigerator. We collected the kidneys and stored them as required for subsequent experiments. All our operations met the requirements of animal ethics and passed the animal ethics approval of Ningbo University. 

***Renal function assessment***

We used specific commercially available kits to detect plasma kidney injury molecule (pKIM-1) (Jiancheng Co., Nanjing, China), plasma neutrophil gelatinase-associated apolipoprotein (pNGAL) (Jiancheng Co., Nanjing, China), and serum creatinine (SCr) (Jiancheng Co., Nanjing, China). 

***Masson staining***

We fixed the kidney tissue with 10% formalin, embedded it in paraffin, sliced it, stained it with Masson, and observed the slice under an optical microscope. According to our previous experimental protocol, the pathological score of kidney tubular epithelial tissue ranged from 0 to 4, as follows: normal histology, score 0; swelling of renal tubular cells, loss of brush border, nuclear condensed, the nuclear loss is less than 25%, score 1; the nuclear loss is between 25% and 50%, score 2; the nuclear loss is between 50% and 75%, score 3; the nuclear loss is more than 75%, score 4 (10). 

***Cell culture***

We cultured KETCs (Boster Biotechnology Co., Ltd., Wuhan, China) in 10% bovine fetal serum. We pretreated the cells with NAC (10 mM), digested the cells with trypsin when the cells were full of culture medium, intervened with LPS for 6 hr, and controlled the reaction speed with DMSO.

***SIRT3 gene silencing and overexpression system***

We used lentiviral transduction (MissionTM shRNA) to silence the KTECs SIRT3 gene *in vitro* and constructed a negative control group (Sigma-Aldrich Co., Shanghai, China). Furthermore, we used Lipofectamine 2000 (Invitrogen Co., Shanghai, China) to transfect KETC cells to construct a pBK-CMV vector (Invitrogen Co., Shanghai, China) to form a SIRT3 gene overexpression system.

***RT-qPCR***

We extracted total RNA from fresh samples and generated cDNA to construct an RT-qPCR reaction system. We used the following sequence amplification: SIRT3 forward: 5’-TGC GTC GTA ACT GCG ACT CC-3’, reverse: 5’-ATC ACT CTG CCT ACA GAA CG-3’; Bcl-2 forward: 5’-CAC CTC CTA AGT CAG CCA GC-3’, reverse: 5’-CAC GCA CCG CAT CCG CAC CA-3’; GAPDH, forward: 5’-CAT CCA TGA CCA CTG ACC TC-3’, reverse: 5’-CGC TCT TGA CGC TGG CAT CA-3’. We set the PCR reaction conditions as follows: 92 °C 12 min, 50 cycles, 96 °C 12 sec, 50 °C 2 min. We used GAPDH standardization and used the 2^-^^△△^^Ct^ method to calculate the relative expression of mRNA.

***Immunochemistry***

We deparaffinized renal cortex tissue sections, added antigen retrieval solution, heated in a microwave oven for 15 min, and gradually cooled to room temperature; immersed the slices in phosphate-buffered saline (PBS) solution, 3% deionized water eliminated active peroxidase, washed with PBS solution; added goat serum working solution dropwise and incubated at room temperature for 20 min; dropwise added primary antibody of inducible nitric oxide synthase (iNOS, Zhenhai Biotechnology Co., Ltd., Ningbo, China), store at -4 °C, and added protein labeling reagent to develop color. Positive cell analysis was performed using an optical density chemiluminescence imaging and analysis system (Azure Biosystems Inc., Dublin, USA).

***Determination of the volume of mitochondria***

We used glutaraldehyde to fix the tissue, stained and observed under a transmission electron microscope (Phillips Co., Tokyo, Japan). The digitized mitochondrial density (Nv, n/μm3) was superimposed on the digitized electron microscope image of the proximal tubule by an orthogonal grid. 

***Western-blotting***

Kidney tissue or KETCs were directly lysed with RIPA (Roche Co., Basel, Switzerland) containing protease and phosphatase inhibitors, the protein was denatured and transferred to the nitrocellulose membrane, incubated with antibodies against toll-like receptor 4 (TLR4) (Santa Cruz Co., CA, US), inhibitor of Kappa Bα (IκBα) (Santa Cruz Co., CA, US), inhibitor of kappa B kinase (IKKβ) (Santa Cruz Co., CA, US), p65 (Santa Cruz Co., CA, US), adenine nucleotide translocator (ANT) (Santa Cruz Co., CA, US), cyclophilin (CypD) (Santa Cruz Co., CA, US) and voltage-dependent anion channel (VDAC) (Santa Cruz Co., CA, US) or GAPDH (Boster Co., Wuhan, China) overnight at 4 ℃, and incubated with anti-rabbit IgG (Cell Signaling Co., Denvers, US) for 1 hr. We washed the membrane and detected the gray values using a gel-image analysis system (Bio-Rad Co., Hercules, US). The gray values were standardized to GAPDH. 

***TdT-mediated dUTP nick-end labeling (TUNEL)***

We took the embedding paraffin and sliced and dyed it. We randomly selected 3 fields of view on each slice to count apoptotic cells and calculated the percentage of apoptotic cells.

***Apoptosis assay***

We used the AnnexinV-FITC/PI kit (Boster Co., Wuhan, China) to evaluate the apoptosis of KTECs, resuspended KTECs and added Annexin V-FITC, incubated for 25 min, and added PI for staining. We used flow cytometry to analyze apoptotic cells and calculated the percentage of apoptotic cells. 

***Enzyme-linked immunosorbent assay (ELISA)***

According to the kit instructions, we used an ELISA kit (Boster Co., Wuhan, China) to determine serum TNF-α and IL-1β levels.

***Statistical analysis***

We expressed the data as mean±standard and used Graphpad Prism 8.0 software for data analysis. We used one-way analysis of variance to compare differences between multiple groups and used Tukey’s *post hoc* test to determine differences between two groups. We used Spearman correlation analysis to analyze SIRT3 mRNA and kidney function parameters. *P*<0.05 was considered a significant difference.

## Results

***Protective effect of SIRT3 on SAKI***

**Figure 1:**
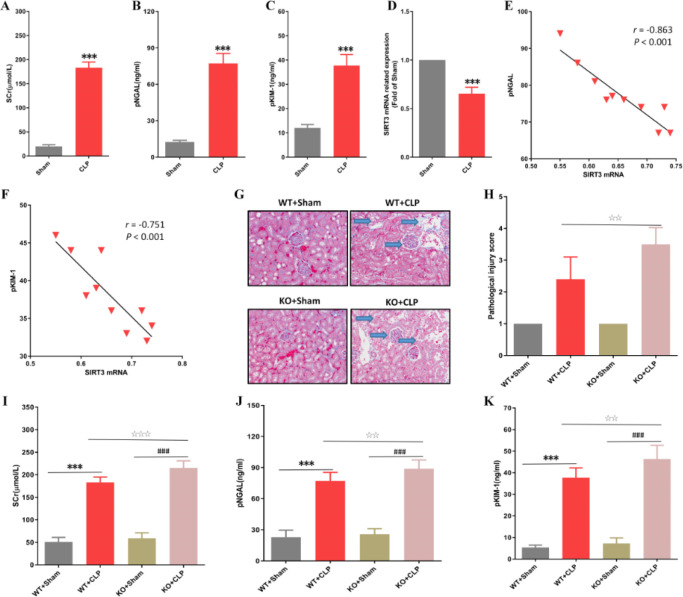
The effect of SIRT3 gene deficiency on acute kidney injury in septic mice

To clarify the effect of SIRT3 on SAKI, we first evaluated the kidney function indexes of mice. We found that Scr, pNGAL, and pKIM-1 induced by CLP were all increased, while the level of SIRT3 mRNA in kidney tissue was decreased (Figure 1, A-D). Our Spearman correlation analysis results suggested that SIRT3 mRNA expression was negatively correlated with the levels of pNGAL and pKIM-1 (Figure 1, E, F). Then we investigated the effect of SIRT3 deficiency on kidney injury, and we found that CLP-induced KTEC degeneration increased, and a large number of neutrophils infiltrated around the glomeruli and interstitium (Figure 1 G). Our results of the semi-quantitative analysis showed that SIRT3 deficiency caused the pathological injury score of kidney tubular epithelial tissue significantly higher (Figure 1 H). Moreover, Scr, pNGAL, and pKIM-1 elevated levels induced by CLP in the SIRT3 KO mice were significantly higher than those in the WT group (Figure 1, I-K). Our results suggested that SIRT3 deficiency could lead to CLP-induced deterioration of kidney function and pathology. 

***SIRT3 protects the mitochondrial function of KTECs***

**Figure 2:**
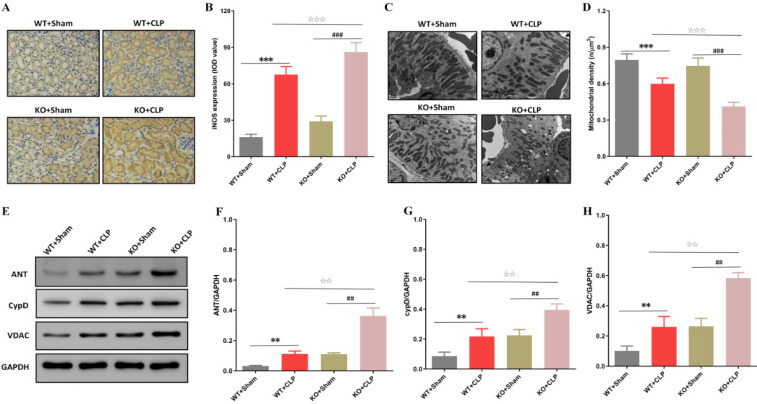
The effect of SIRT3 on mitochondrial function in KTECs of septic mice

Previous studies indicated that SIRT3 has a significant protective effect on mitochondrial function, but the impact of SIRT3 on the mitochondrial structure and function of KTECs has not been clarified (11, 12). Therefore, we compared SIRT3 KO and WT groups, and we found that CLP-induced iNOS production and mitochondrial density reduction in kidney tissue of the KO group was increased (Figure 2. A-D). Moreover, we detected the expression of mitochondrial permeability transition pore (mPTP)-related proteins, and our results showed that SIRT3 deficiency caused an increase in the expression of ANT, CypD, and VDAC in the kidney tissue (Figure 2, E-H). All our results suggested that SIRT3 deficiency enhanced the production of iNOS and changes in mitochondrial function related to kidney injury, and it was clear that the protective effect of SIRT3 on SAKI was related to the changes in mitochondrial mPTP function.

***Effect of SIRT3 deficiency on inflammatory response of SAKI***

**Figure 3:**
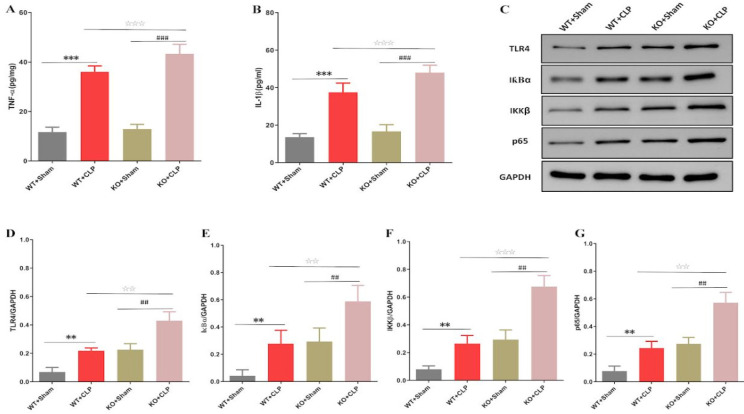
The effect of SIRT3 gene deletion on renal inflammatory response in septic mice

To explore the inflammatory regulation of SIRT3 in mice with SAKI, we detected the changes of TNF-α and IL-1β in kidney tissue. Our results showed that SIRT3 deficiency caused a decrease in TNF-α and IL-1β (Figure 3. A, B). In addition, we evaluated the expression of TLR4-NF-κB protein, and we found that CLP induced the up-regulation of TLR4, NF-κB IκBα, IKKβ, and p65 protein expression, and this effect in SIRT3 KO group was greater than in WT group (Figure 3, C-G). Therefore, SIRT3 protected SAKI by inhibiting TLR4-NF-κB.

***NAC protects SAKI by inhibiting TLR4-NF-κB***

**Figure 4:**
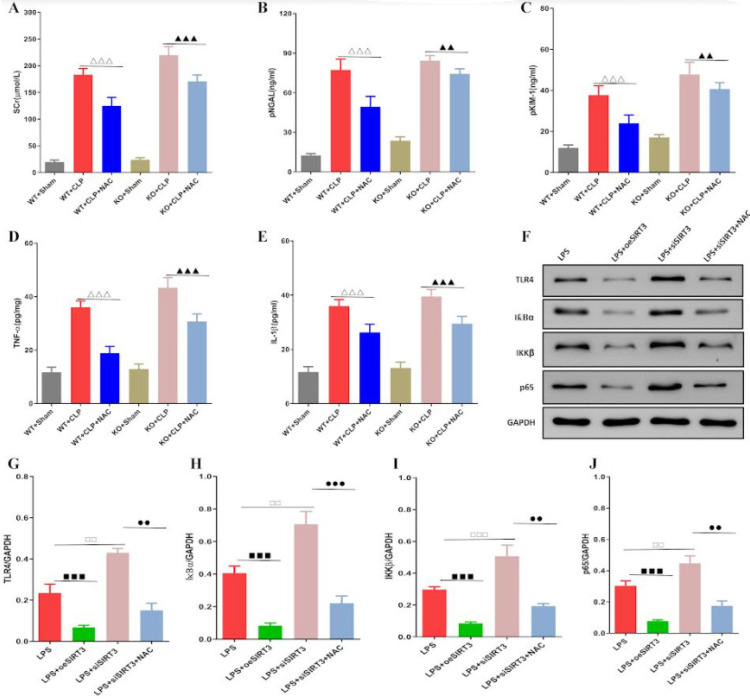
NAC protects SAKI by inhibiting TLR4-NF-κB signal pathway

Based on the oxidative stress in the kidneys of mice during SAKI, we explored the therapeutic effect of anti-oxidant NAC on SAKI mice. We found that NAC significantly reduced Scr, pNGAL, and pKIM-1 induced by CLP in WT and SIRT3 KO groups, while NAC also reduced TNF-α and IL-1β in both groups (Figure 4, A-E). To further identify the impact of SIRT3 on SAKI and the protective mechanism of NAC, we constructed an overexpression and silencing system of SIRT3 in KTECs. The expression of TLR4, NF-κB IκBα, IKKβ, and p65 proteins induced by LPS in KTECs was significantly down-regulated after SIRT3 gene overexpression. On the contrary, LPS-induced TLR4, NF-κB IκBα, IKKβ, and p65 protein expression were all significantly up-regulated after SIRT3 silencing. Meanwhile, NAC could down-regulate these protein expressions in KTECs induced by LPS after SIRT3 silencing (Figure 4, F-J). It could be seen that NAC had a significant protective effect on SAKI by inhibiting TLR4-NF-κB. 

***NAC inhibits apoptosis of KTECs by up-regulating SIRT3 expression***

**Figure 5:**
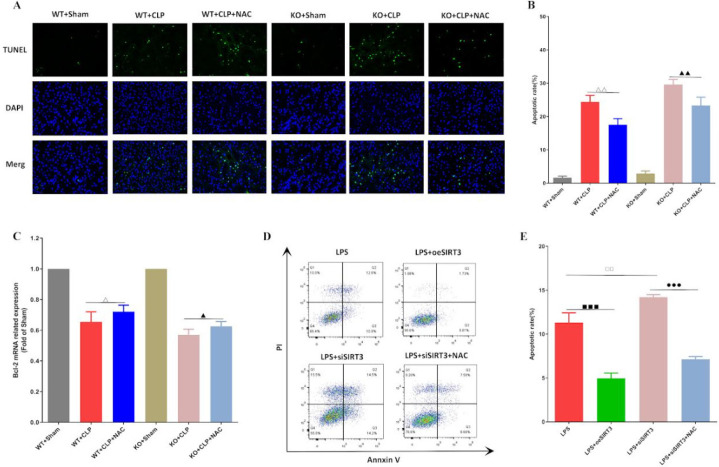
NAC inhibits sepsis induced KETCs apoptosis by upregulating SIRT3 expression

Apoptosis plays a key role in SAKI, but the specific mechanism of SIRT3 regulating apoptosis of KTECs and the protective effect of NAC on apoptosis of KTECs have not yet been elucidated. Therefore, we detected the apoptosis of KTECs and assessed the impact of NAC on the LPS-induced KTECs apoptosis. CLP induced increased KTEC apoptosis in the SIRT3 KO group, and NAC could significantly inhibit the apoptosis of kidney tissue (Figure 5; A, B). Moreover, we detected the level of anti-apoptotic protein Bcl-2 mRNA in kidney tissue. Consistent with the above results, we found that the level of Bcl-2 mRNA in the SIRT3 KO+CLP group was reduced, and NAC could partially restore the expression of Bcl-2 mRNA (Figure 5 C). Moreover, we constructed a KTECs cell system with SIRT3 overexpression and silence and detected the apoptosis of KTECs. We found that SIRT3 silencing enhanced LPS-induced apoptosis, and NAC could significantly inhibit the number of apoptosis. In contrast, overexpression of SIRT3 significantly inhibited LPS-induced KTEC apoptosis (Figure 5 D, E). These suggested that SIRT3 deficiency could promote KTECs apoptosis, while NAC could partially restore this effect.

(A-C) Scr, pNGAL, and pKIM-1 levels. (D) Relative expression of SIRT3 mRNA. (E) Correlation between SIRT3 mRNA expression and pNGAL level. (F) Correlation between SIRT3 mRNA expression and pKIM-1 level. (G) Renal pathology (Masson, ×200). (H) Renal pathological scores. (I-K) Scr, pNGAL, and pKIM-1 levels. The data was expressed as mean±standard. ***P<0.001 vs Sham; ###P<0.001 vs the KO+Sham; *P*<0.01, *P*<0.001 vs the WT+CLP.

SIRT3: sirtuin 3; SAKI: septic acute kidney injury; Scr: Serum creatinine; pNGAL: plasma neutrophil gelatinase-associated lipocalin; pKIM-1: plasma kidney injury molecule-1

(A-B) iNOS protein expression (IHC, ×400). (C) Mitochondrial structure changes of KTECs. (D) Average mitochondrial density (n/μm3). (E) Mitochondrial mPTP-related protein expression. (F-H) ANT, CypD, and VDAC proteins relative expression. The data were expressed as mean±standard. **P<0.01, ***P<0.001 vs the WT+Sham; ##*P*<0.01, ###*P*<0.001 vs KO+Sham; *P*<0.01, *P*<0.001 vs WT+CLP.

SIRT3: sirtuin 3; KTECs: kidney tubular epithelial cells; iNOS: inducible nitric oxide synthase

(A-B) TNF-α and IL-1β levels. (C) TLR4-NF-κB expression in the kidney. (D-G) TLR4, IκBα, IKKβ, and p65 protein levels. The data were expressed as mean±standard. **P<0.01, ***P<0.001 vs WT+Sham; ##*P*<0.001, ###*P*<0.001 vs KO+Sham; *P*<0.01, *P*<0.001 vs WT+CLP.

SIRT3: sirtuin 3; SAKI: septic acute kidney injury; TLR4: toll-like receptor 4; NF-κB: nuclear factor-kappa B

(A-C) Effect of NAC on Scr, pNGAL, and pKIM-1. (D) Effect of NAC on TNF-α. (E) Effect of NAC on IL-1β. (F) NAC regulates TLR4-NF-κB by partially restoring SIRT3 in KTECs. (G-J) TLR4, IκBα, IKKβ, p65 protein levels. The data was expressed as mean±standard. *P*<0.001 vs the WT+CLP; *P*<0.01, *P*<0.001 vs the KO+CLP; ■■■*P*<0.001, □□*P*<0.01, *P*<0.001 vs the LPS;*P*<0.01, *P*<0.001 vs the LPS+siSIRT3. NAC: N-acetylcysteine; SIRT3: sirtuin 3; SAKI: septic acute kidney injury; KTECs, kidney tubular epithelial cells.

(A) NAC affects the apoptosis of KETCs. (B) Apoptotic rate of KETCs. (C) Bcl-2 mRNA expression. (D) NAC inhibits apoptosis. (E) Apoptotic rate of KETCs. The data was expressed as mean±standard. *P*<0.05, *P*<0.01 vs the WT+CLP; *P*<0.05, *P*<0.01 vs the KO+CLP; ■■■*P*<0.001, □□*P*<0.01 vs the LPS; *P*<0.05, *P*<0.01 vs the LPS+siSIRT3. NAC: N-acetylcysteine; SIRT3: sirtuin 3; SAKI: septic acute kidney injury; KTECs, kidney tubular epithelial cells.

## Discussion

AKI is one of the most common clinical critical illnesses, which can be caused by sepsis, nephrotoxic drugs, and renal ischemia, and its incidence in critically ill patients is as high as 25% (1). There are various mechanisms of AKI, and mitochondrial injury is one of the important pathophysiological signs (13). AKI mainly involves the injury of KTECs, which are rich in mitochondria. Therefore, the change in mitochondrial dysfunction in KTECs is an important sign of AKI. SIRT3 is a highly conserved deacetylase that relies on nicotinamide adenine dinucleotide, and it participates in the production, fusion, division, and autophagy of mitochondria (14). SIRT3 exists in the form of a long chain, which has a mitochondrial-related targeting sequence located at N-terminus. When oxidative stress and other stimuli occur, the long-chain SIRT3 in the cell is hydrolyzed by matrix processing peptidase to remove its N-terminal mitochondrial sequence into short-chain SIRT3 and enter the mitochondria to function (15). 

According to reports, more than 65% of mitochondrial protein transformation and modification need to be completed through acetylation (16). Mitochondrial proteins are highly acetylated in liver tissue, brown adipose tissue, and myocardial tissue after SIRT3 KO, and this change occurs simultaneously with the down-regulation of SIRT3 expression (17). It can be seen that SIRT3 can regulate the level of mitochondrial protein acetylation, thereby regulating body metabolism (18). In the present study, SIRT3 deficiency resulted in decreased renal function, damage to KTECs, and increased apoptosis. Our experimental results are consistent with Morigi *et al*. (19). SIRT3 decrease is related to the abnormality of KTECs mitochondria, which can aggravate cisplatin cause AKI, and the increase of SIRT3 level can partially improve kidney function (19).

In the present study, we built a mice model through CLP and found that SIRT3 deletion induced an increase in ROS production in KTECs, and at the same time increased mitochondrial damage. This phenomenon suggested that SIRT3 acted as a KTECs protective agent against oxidative stress damage and mitochondrial dysfunction. Our results confirmed that SIRT3 modulated the production of ROS in KTECs through deacetylation modification and regulated the activity of related oxidases, thereby protecting kidney function (20). In addition, studies indicated that SIRT3 can reduce ischemia-reperfusion injury and protect mitochondrial function by activating the anti-oxidant enzymes, suggesting that SIRT3 can be used to treat AKI ischemia-reperfusion injury (21).

The energy demand of cells depends on the dynamic balance between mitochondrial fission and fusion, while this normal balance is affected by various internal and external factors. According to reports, SIRT3 can reduce the aggregation of KTECs motility-related protein 1 in mitochondria through deacetylation, and it is confirmed that SIRT3 can restrict mitochondrial division by regulating the expression of motility-related protein 1 and the split protein on the outer mitochondrial membrane (22). Other studies indicated that SIRT3 participates in calcium ion dynamics and is related to the regulation of cell apoptosis (23, 24). Apoptosis depends on mPTP, and continuous opening of the mPTP will lead to the pro-apoptotic pathway activation, cytochrome C release, and mitochondrial membrane potential loss (25). 

In the present study, our results showed that SIRT3 deficiency induced the increased expression of mPTP-related proteins ANT, CypD, and VDAC in KTECs, and a significant increase in iNOS production, indicating that SIRT3 deficiency enhanced iNOS production, and mitochondrial function changed especial mitochondrial mPTP function changes. Furthermore, we also found that SIRT3 deficiency caused a significant increase in renal inflammation and apoptosis, while TLR4, NF-κB, IKKβ, IκBα, and p65 proteins were significantly up-regulated. Anti-oxidant NAC could significantly inhibit SAKI inflammatory response and apoptosis, which had a significant protective effect on SAKI. Studies showed that SIRT3 can prevent the continuous opening of mPTP through deacetylation of cytochrome D, thereby preventing the production of reactive oxygen species, stabilizing calcium ion dynamics, stabilizing mitochondrial dynamics, and reducing cell apoptosis (26, 27). Therefore, SIRT3 is an important protein that regulates mitochondrial dynamics.

## Conclusion

Our study found that SIRT3 had a significant protective effect on SAKI by inhibiting oxidative stress, apoptosis, and inflammatory cytokines. NAC protected SAKI by inhibiting SIRT3-mediated mitochondrial dysfunction and apoptosis of KTECs. Therefore, above results revealed the protective mechanism of the SIRT3 and highlighted the potential therapeutic effect of NAC on SAKI. 

## Authors’ Contributions

H F, JW L, MS, and JH Z performed the experiments, interpreted data, and wrote the first draft of the manuscript. HF and JH Z participated in conception, design, and critical revisions. All authors read and approved the final manuscript.

## Conflicts of Interest

No conflicts of interest to declare. 

## References

[1] Ronco C, Bellomo R, Kellum JA (2019). Acute kidney injury. Lancet.

[2] Fan H, Le JW, Sun M, Zhu JH (2021). Sirtuin 3 deficiency promotes acute kidney injury induced by sepsis via mitochondrial dysfunction and apoptosis. Iran J Basic Med Sci.

[3] Jacob J, Dannenhoffer J, Rutter A (2020). Acute kidney injury. Prim Care.

[4] Fan H, Le JW, Zhu JH (2020). Protective Effect of N-acetylcysteine pretreatment on acute kidney injury in septic rats. J Surg Res.

[5] Bellomo R, Kellum JA, Ronco C, Wald R, Martensson J, Maiden M (2017). Acute kidney injury in sepsis. Intensive Care Med.

[6] Zhao WY, Zhang L, Sui MX, Zhu YH, Zeng L (2016). Protective effects of sirtuin 3 in a murine model of sepsis-induced acute kidney injury. Sci Rep.

[7] Klimova N, Fearnow A, Long A, Kristian T (2020). NAD+precursor modulates post-ischemic mitochondrial fragmentation and reactive oxygen species generation via SIRT3 dependent mechanisms. Exp Neurol.

[8] Wang Z, Sun R, Wang G, Chen Z, Li Y, Zhao Y (2020). SIRT3-mediated deacetylation of PRDX3 alleviates mitochondrial oxidative damage and apoptosis induced by intestinal ischemia/reperfusion injury. Redox Biol.

[9] Wei S, Gao Y, Dai X, Fu W, Cai S, Fang H (2019). SIRT1-mediated HMGB1 deacetylation suppresses sepsis-associated acute kidney injury. Am J Physiol Renal Physiol.

[10] Fan H, Zhao Y, Zhu JH (2019). S-nitrosoglutathione protects lipopolysaccharide-induced acute kidney injury by inhibiting toll-like receptor 4-nuclear factor-κB signal pathway. J Pharm Pharmacol.

[11] Zhao W, Zhang L, Chen R, Lu H, Sui M, Zhu Y (2018). SIRT3 protects against acute kidney injury via AMPK/mTOR-regulated autophagy. Front Physiol.

[12] Perico L, Morigi M, Benigni A (2016). Mitochondrial Sirtuin 3 and Renal Diseases. Nephron.

[13] Perico L, Remuzzi G, Benigni A (2015). Sirtuin 3 in acute kidney injury. Oncotarget.

[14] Li Y, Ye Z, Lai W, Rao J, Huang W, Zhang X (2017). Activation of sirtuin 3 by silybin attenuates mitochondrial dysfunction in cisplatin-induced acute kidney injury. Front Pharmacol.

[15] Xu S, Gao Y, Zhang Q, Wei S, Chen Z, Dai X (2016). SIRT1/3 activation by resveratrol attenuates acute kidney injury in a septic rat model. Oxid Med Cell Longev.

[16] Peerapanyasut W, Kobroob A, Palee S, Chattipakorn N, Wongmekiat O (2019). N-acetylcysteine attenuates the increasing severity of distant organ liver dysfunction after acute kidney injury in rats exposed to bisphenol A. Antioxidants (Basel).

[17] Allison SJ (2015). Acute kidney injury: Sirtuin 3-a master regulator of mitochondrial integrity in AKI. Nat Rev Nephrol.

[18] Huang YT, Chen YY, Lai YH, Cheng CC, Lin TC, Su YS (2016). Resveratrol alleviates the cytotoxicity induced by the radiocontrast agent, ioxitalamate, by reducing the production of reactive oxygen species in HK-2 human renal proximal tubule epithelial cells in vitro. Int J Mol Med.

[19] Morigi M, Perico L, Benigni A (2018). Sirtuins in renal health and disease. J Am Soc Nephrol.

[20] Morigi M, Perico L, Rota C, Longaretti L, Conti S, Rottoli D (2015). Sirtuin 3-dependent mitochondrial dynamic improvements protect against acute kidney injury. J Clin Invest.

[21] Ouyang J, Zeng Z, Fang H, Li F, Zhang X, Tan W (2019). SIRT3 Inactivation promotes acute kidney injury through elevated acetylation of SOD2 and p53. J Surg Res.

[22] Yuan Y, Zhu L, Li L, Liu J, Chen Y, Cheng J (2019). S-sulfhydration of sirt3 by hydrogen sulfide attenuates mitochondrial dysfunction in cisplatin-induced acute kidney injury. Antioxid Redox Signal.

[23] Wang Q, Xu J, Li X, Liu Z, Han Y, Xu X (2019). Sirt3 modulate renal ischemia-reperfusion injury through enhancing mitochondrial fusion and activating the ERK-OPA1 signaling pathway. J Cell Physiol.

[24] Li M, Li CM, Ye ZC, Huang J, Li Y, Lai W (2020). Sirt3 modulates fatty acid oxidation and attenuates cisplatin-induced AKI in mice. J Cell Mol Med.

[25] Huang Z, Li Q, Yuan Y, Zhang C, Wu L, Liu X (2019). Renalase attenuates mitochondrial fission in cisplatin-induced acute kidney injury via modulating sirtuin-3. Life Sci.

[26] Zhang Q, Liu X, Li N, Zhang J, Yang J, Bu P (2018). Sirtuin 3 deficiency aggravates contrast-induced acute kidney injury. J Transl Med.

[27] Tan C, Gu J, Li T, Chen H, Liu K, Liu M (2021). Inhibition of aerobic glycolysis alleviates sepsis-induced acute kidney injury by promoting lactate/Sirtuin 3/AMPK-regulated autophagy. Int J Mol Med.

